# Fully equipped CARs to address tumor heterogeneity, enhance safety, and improve the functionality of cellular immunotherapies

**DOI:** 10.3389/fimmu.2024.1407992

**Published:** 2024-06-03

**Authors:** Antonio Maria Alviano, Marta Biondi, Erica Grassenis, Andrea Biondi, Marta Serafini, Sarah Tettamanti

**Affiliations:** ^1^ Tettamanti Center and Pediatrics, Fondazione IRCCS San Gerardo dei Tintori, Monza, Italy; ^2^ School of Medicine and Surgery, University of Milano-Bicocca, Monza, Italy

**Keywords:** CAR-T cells, adapter CARs (AdCARs), tandem CARs (TanCARs), logic-gated CARs, safety switches, epitope editing, antigen density

## Abstract

Although adoptive transfer of chimeric antigen receptor (CAR)-engineered T cells has achieved unprecedented response rates in patients with certain hematological malignancies, this therapeutic modality is still far from fulfilling its remarkable potential, especially in the context of solid cancers. Antigen escape variants, off-tumor destruction of healthy tissues expressing tumor-associated antigens (TAAs), poor CAR-T cell persistence, and the occurrence of functional exhaustion represent some of the most prominent hurdles that limit CAR-T cell ability to induce long-lasting remissions with a tolerable adverse effect profile. In this review, we summarize the main approaches that have been developed to face such bottlenecks, including the adapter CAR (AdCAR) system, Boolean-logic gating, epitope editing, the modulation of cell-intrinsic signaling pathways, and the incorporation of safety switches to precisely control CAR-T cell activation. We also discuss the most pressing issues pertaining to the selection of co-stimulatory domains, with a focus on strategies aimed at promoting CAR-T cell persistence and optimal antitumor functionality.

## Highlights

- CAR-T cell therapy has significantly transformed immuno-oncology; however, there are still several challenges that need to be addressed.
- Adapter CARs and “OR” logic-gating allow to improve CAR-T cell activity against heterogeneous tumors.
- “AND” logic-gating strategies and ON/OFF switches can be implemented to enhance CAR-T cell safety.
- Careful selection of co-stimulatory domains and targeted manipulations of intracellular signaling pathways allow for the optimization of CAR-T cell functionality.

## 1 Background

The outstanding results achieved by immunotherapy in patients with malignant neoplasms have fueled a growing interest in this approach to cancer treatment, which aims to harness the potential of the immune system to target neoplastic diseases. Among passive immunotherapeutic approaches, chimeric antigen receptor (CAR)-T cell therapy has emerged as an extremely powerful tool, particularly in the context of relapsed/refractory (R/R) B-cell malignancies and multiple myeloma, leading to the approval of six CAR-T cell products (as of early 2024) by the Food and Drug Administration (FDA) and the European Medicines Agency (EMA) (1, 2). CAR-T cells are genetically engineered T lymphocytes expressing artificial receptors, which typically combine the single-chain variable fragment (scFv) derived from a monoclonal antibody (mAb) with the intracellular signaling machinery of T cells. The goal is to elicit T-cell effector functions and cytotoxic activity upon the recognition of the selected target antigen (3). While the intracellular portion of first-generation CARs consists solely of the T-cell receptor (TCR) CD3ζ signaling domain, second- and third-generation CARs also contain one or two co-stimulatory domains, respectively (4).

Even though CAR-T cell therapy has significantly transformed immuno-oncology, its broader application to various hematological malignancies and solid tumors has encountered many obstacles. These challenges include, among others, tumor heterogeneity, lack of cancer-specific antigens (with the subsequent risk of on-target-off-tumor toxicities), and limited CAR-T cell expansion and persistence (5, 6).

Therefore, several strategies have been proposed to prevent antigen escape, improve CAR-T selectivity for malignant cells, and promote their long-term persistence (7).

## 2 Tackling tumor heterogeneity

**Figure 1:**
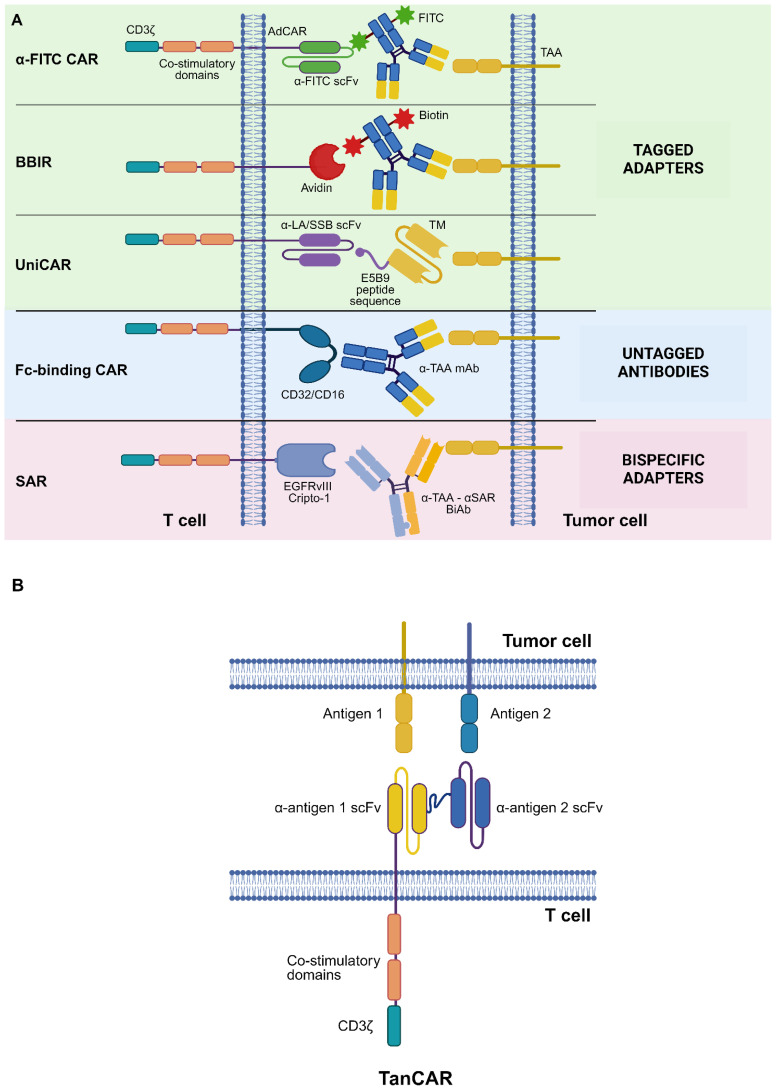
CAR design strategies to tackle tumor heterogeneity. **(A)** Adapter CAR (AdCAR) platform. Shown are tagged adapters, untagged antibodies (Abs), and bispecific adapters. **(B)** “OR” logic-gating: TanCAR system. See Glossary for abbreviations. Modified from Refs. 7, 10. Figure created with BioRender.com.

A high degree of genomic instability is a hallmark of cancer, leading to the continuous accumulation of genetic and epigenetic alterations throughout the natural history of the disease (8). The emergence of genetically and phenotypically distinct malignant subclones can have a crucial role in tumor invasiveness, dissemination, and resistance to treatment. In this context, the selective pressure imposed by conventional single-targeted CAR-T cells could favor the development of antigen-negative neoplastic subpopulations, resulting in disease relapse. Clinical data regarding patients with B-cell malignancies treated with CD19.CAR-T cells indicate that up to 50% of them relapse within 12 months of initial infusion, and that antigen loss, downregulation or modulation represent pivotal mechanisms underlying treatment failure (9). To overcome these drawbacks, a considerable body of research has been focusing on next-generation engineering approaches, such as the adapter CAR (AdCAR) platform and the “OR” logic-gating system, allowing for the simultaneous or sequential targeting of different antigens by CAR-T cells (**Figure 1**).

### 2.1 Adapter CAR-T cells

In the adapter CAR (AdCAR) system, T lymphocytes are transduced with a second- or third-generation CAR whose antigen-binding domain recognizes an exogenous adapter molecule (AM) rather than a membrane protein expressed on target cells. Adapters are bifunctional molecules with distinct binding domains, which allow them to bridge target antigen-expressing cells with AdCAR-T cells (10) (**Figure 1A**).

A key advantage of the AdCAR platform lies in the possibility of targeting multiple antigens with a single CAR-T cell product. The significant degree of flexibility of this therapeutic strategy allows to rapidly redirect the specificity of CAR-T cells towards a different target antigen by simply switching the AM. This approach is considerably more cost-effective and time-saving compared to re-isolating T lymphocytes, transducing them with a newly designed CAR, and performing a second CAR-T cell infusion. Another favorable characteristic of this design is that it allows to implement treatment-free intervals in the therapeutic protocol by simply withholding the AM. This, in turn, safeguards T-lymphocytes from exhaustion induced by chronic stimulation without the need for CAR-T cell depletion strategies.

Moreover, the AdCAR platform is precisely controllable, since the clearance of the AM leads to the cessation of CAR-T cell activity. In addition, modulating the concentration of the AM at the tumor site allows to fine-tune the magnitude of CAR-T cell response. Thus, it is possible to achieve satisfactory tumor control while avoiding untoward effects associated with T-cell hyperactivation (e.g., cytokine release syndrome [CRS]). In this regard, the AdCAR system can be considered a form of safety switch (specifically, a “killing switch”), allowing regulated CAR-T cell activation (11). Other types of switches specifically conceived to enhance CAR-T cell safety will be discussed in the following sections.

Potential limitations of the AdCAR approach are related to the fact that AdCAR-T cells are functionally inert in the absence of the corresponding AMs. For this reason, the AM concentration at the tumor site needs to be maintained above the threshold required for an efficient CAR-T cell – tumor cell bridging for an adequate amount of time. Moreover, some of the extracellular domains that can be incorporated into the AdCAR structure, particularly avidin and streptavidin, are immunogenic. Lastly, the use of Fc-glycoengineered antibodies (Abs) as AMs has been associated with the occurrence of CRS (12).

Three main classes of AMs are recognized, namely tagged adapters, untagged immunoglobulins (Igs), and bispecific adapters (10).

#### 2.1.1 Tagged adapters

Tagged adapters can be labeled with various molecules, each of which allows them to bind the extracellular domain of an aptly designed AdCAR.

Anti-fluorescein isothiocyanate (FITC) AdCAR-T cells in combination with FITC-labeled rituximab demonstrated a remarkable antitumor efficacy in an immunocompetent mouse model of rituximab-resistant B-cell non-Hodgkin lymphoma (NHL) (13).

Similarly, it has been shown that FITC-labeled trastuzumab allows to redirect AdCAR-T cells against epidermal growth factor receptor 2 (HER2)-expressing breast cancer cells, resulting in the eradication of established tumors in xenograft mouse models (14).

Biotinylated adapters have been employed in conjunction with AdCARs whose antigen-binding domain consists of avidin or streptavidin: biotin-binding immune receptor (BBIR) T cells in combination with biotinylated anti-mesothelin (MSLN) Abs have been shown to possess a potent antitumor activity against MSLN^+^ ovarian cancer, both *in vitro* and *in vivo* (15).

Nixdorf and colleagues designed and tested a biotin-directed AdCAR in combination with biotinylated anti-CD33, anti-human C-type lectin-like molecule-1 (CLL-1), or anti-CD123 AMs against acute myeloid leukemia (AML) cell lines and primary AML (pAML), both *in vitro* and *in vivo*. AdCAR-T cells demonstrated a specific cytotoxic activity when co-cultured with AML cell lines and pAML cells. Interestingly, their cytolytic potential and later effector functions were markedly improved by the intermittent rather than continuous stimulation with AMs, possibly due to a transcriptional reprogramming mechanism. This reprogramming resulted in an enhanced expression of memory-related genes during periods of functional rest, with the subsequent re-expression of activation markers upon restimulation. Furthermore, the sequential administration of AMs of different specificities was found to significantly improve tumor control by AdCAR-T cells in a long-term co-culture with pAML blasts, underscoring the potential of the AdCAR strategy to target heterogeneous tumors (16).

Target modules (TMs) are AMs tagged with a short peptide sequence, such as the E5B9 epitope of the La/SS-B nuclear protein, which is recognized by an AdCAR bearing an anti-La scFv as its extracellular domain: the corresponding AdCAR platform, known as the “UniCAR” system, has shown promise in preclinical models of various malignancies, including AML, B-cell NHL, disialoganglioside GD2^+^ neuroblastoma, and sialyl-Tn (STn)^+^ breast cancer (17–22).

A further development of the “UniCAR” platform involves the use of a trimeric TM consisting of an antigen-binding domain, the E5B9 epitope, and a single-chain format of the 4-1BB ligand (4-1BBL). The latter is the natural ligand of the 4-1BB co-stimulatory receptor, which plays a crucial role in eliciting the “second signal” required for T-cell activation. The incorporation of the 4-1BBL into the structure of TMs provides UniCAR-T cells with transient co-stimulation at the tumor site, potentially boosting their effector functions. Indeed, a preclinical study focusing on AML has shown that trimeric TMs directed against CD123 (TM123-4-1BBL) improve the persistence and long-term cytolytic capabilities of UniCAR-T cells as compared to conventional TM123 modules both *in vitro* and *in vivo*. An additional advantage of trimeric TMs was identified in their prolonged terminal plasma half-life, due to their higher hydrodynamic volume as compared to TM123 modules. This, in turn, could allow for more sustained anticancer responses by UniCAR-T cells. Of note, co-culture experiments with the MOLM-13 AML line showed that a significant cytokine secretion by UniCAR-T cells is elicited only at concentrations of the TM123-4-1BBL module much higher than those required for tumor cell killing. This could have important safety implications, as UniCAR-T cells might eradicate bulky tumors without causing cytokine-mediated adverse effects (23).

Interestingly, tagged adapters can also be employed to selectively modulate the functional properties of AdCAR-T cells without affecting bystander (CAR^-^) cells. Luo and colleagues demonstrated that a FITC-labeled toll-like receptor-7 agonist (FITC-TLR7a) can restore the antitumor activity and proliferative capacities of exhausted FITC.CAR-T cells both *in vitro* and *in vivo* without significant toxicities. The proposed mechanism involves internalization of the FITC-TLR7a – CAR complex via receptor-mediated endocytosis. This is followed by TLR-7 activation in the endosomal compartment of AdCAR-T cells (24).

#### 2.1.2 Untagged immunoglobulins

Untagged Abs can function as AMs for AdCARs bearing the extracellular portion of FcγRIII (CD16) or FcγRII (CD32) as their antigen-binding domain (25).

CD16-based AdCAR-T cells engineered to express a high-affinity CD16 variant (CD16V-BB-ζ T lymphocytes) were shown to be highly cytolytic against the Daudi lymphoma cell line or primary chronic lymphocytic leukemia (CLL) cells in the presence of rituximab; against osteosarcoma or neuroblastoma cells in the presence of an anti-GD2 mAb; or against HER2-expressing stomach and breast cancer cells in combination with trastuzumab. These results were also validated *in vivo*, where CD16V-BB-ζ T cells exerted remarkable antitumor effects in immunodeficient mice engrafted with the NB1691 neuroblastoma cell line (in the presence of an anti-GD2 mAb) or with the Daudi cell line (in combination with rituximab) (26).

Besides modifying the CD16 sequence, it is possible to employ glycoengineered Abs with increased CD16 affinity as AMs. Rataj and colleagues demonstrated that the Fc-modified anti-CD20 Ab obinutuzumab allows to boost the activation and antitumor functionality of CD16.CAR-T cells against the CD20^+^ Raji lymphoma cell line when compared to a non-glycoengineered anti-CD20 Ab recognizing the same epitope. Similarly, in a preclinical model of malignant melanoma, improved tumor cell killing was observed when combining CD16-CAR-T cells with the glycoengineered anti-melanoma-associated chondroitin sulfate proteoglycan (MCSP) Ab LC007 as compared to the corresponding wild-type (WT) Ab (12).

#### 2.1.3 Bispecific adapters

Bispecific adapters are artificial molecules designed to simultaneously bind multiple target antigens. They generally consist of bispecific antibodies (BiAbs) bearing two distinct scFvs, one of which recognizes a given tumor-associated antigen (TAA) and the other binds to a suitable AdCAR.

In the synthetic agonist receptor (SAR) platform, T lymphocytes are transduced with AdCARs bearing the ECD of the Cripto-1 embryonic antigen or the EGFR variant III (EGFRvIII, which is not expressed in normal tissues). Engineered cells are then administered in combination with bispecific adapters that simultaneously bind a designated TAA and either Cripto-1 or EGFRvIII. It has been shown that SAR-T cells, in conjunction with an anti-epithelial cell adhesion molecule (EpCAM) x anti-EGFRvIII BiAb, mediate potent cytotoxic effects upon EpCAM^+^ tumor cell encounter *in vitro*. Moreover, the administration of SAR-T cells together with an anti-MSLN x anti-EGFRvIII BiAb was found to significantly prolong the survival of immunodeficient mice engrafted with MSLN^+^ pancreatic cancer cells, outperforming conventional MSLN.CAR-T cells (27).

#### 2.1.4 Clinical trials evaluating AdCAR-T cells

Given that osteosarcoma cells frequently overexpress folate receptors, the NCT05312411 phase I trial is evaluating the use of second-generation anti-FITC AdCAR-T cells in combination with FITC-conjugated folate as a treatment strategy for R/R osteosarcoma in young adults (28).

The NCT04230265 dose-escalating phase I trial is evaluating the UniCAR-T-CD123 system AVC-101, developed by AvenCell Therapeutics (Cambridge, MA, USA), as a treatment strategy for adult patients with heavily-pretreated, R/R AML or blastic plasmacytoid dendritic cell neoplasm (BPDCN) (29, 30). This platform takes advantage of a universal CAR that recognizes a CD123-specific TM to redirect UniCAR-T cells towards CD123^+^ targets. Preliminary results indicated an acceptable safety profile and a meaningful clinical activity of this UniCAR system, with two complete remissions with incomplete hematologic recovery (CR_i_) and a partial remission (PR) observed among the three patients who completed treatment (31). In subsequent reports, an encouraging clinical efficacy was observed, with an objective response rate (ORR) of 53% in the first 19 AML patients who completed the full treatment course (32, 33).

The UniCAR system is also being tested in solid tumors: the NCT04633148 dose-escalating phase I trial is currently recruiting patients with prostate-specific membrane antigen (PSMA)^+^ prostate cancer to evaluate the safety and efficacy of the PSMA-directed UniCAR platform AVC-102 (also developed by AvenCell Therapeutics) (34).

The CD16-BB-ζ AdCAR-T cell system ACTR087, developed by Cogent Biosciences (Cambridge, MA, USA), has been evaluated in combination with rituximab in patients with R/R B-cell NHL unresponsive to rituximab monotherapy (NCT02776813). In all dose levels (DLs) tested, an ORR of 50% was reported, with an adverse event profile consisting of two cases of severe immune effector cell-associated neurotoxicity syndrome (ICANS) and four cases of severe CRS, leading to the death of three patients (35, 36).

Another CD16-based AdCAR-T cell product (ACTR707, also developed by Cogent Biosciences) has been evaluated in conjunction with trastuzumab in patients with HER2-expressing advanced solid tumors, showing no dose-limiting adverse events (NCT03680560) (37).

Of note, the use of Fc-binding AdCAR-T cells should be avoided in patients with hypergammaglobulinemia, due to the risk of adverse events stemming from T-cell hyperactivation.

### 2.2 “OR” logic-gated CAR-T cells: tandem CARs

The application of Boolean-logic gating to CAR-T cell design has been envisioned as a promising strategy to address tumor heterogeneity as well as to improve CAR-T cell safety (as will be discussed in the next sections) (38). Regarding the first point, the “OR” logic gate allows to target a heterogeneous tumor population and to retain CAR-T cell efficacy even in the face of clonal escape or antigen-negative relapse.

Among “OR” logic-gated platforms, the tandem CAR (TanCAR) system entails the engineering of T lymphocytes to express a CAR with two distinct scFvs connected in tandem via a flexible linker. This strategy allows to trigger a T-cell response upon the engagement of either of the two targeted antigens. Notably, the simultaneous binding of both target antigens elicits an exponentially more potent response by TanCAR-T cells, pointing towards a synergistic effect of double-binding on their cytotoxic capabilities (39) (**Figure 1B**). This bivalency boosts CAR signal strength, possibly resulting in a superior antitumor functionality as compared to, e.g., the AdCAR system.

However, an excessive CAR signal strength could promote CAR-T cell exhaustion, limit their persistence, and increase the risk of adverse events. In addition, this next-generation CAR design is not suitable for targeting TAAs with a significant degree of expression on normal tissues, due to the risk of intolerable off-tumor toxicities. Other limitations are related to the size of the TanCAR transgene, which could exceed the cargo capacity of lentiviral vectors used for transduction, as well as the possibility of cross-linking events involving the light and heavy chain variable (VL-VH) domains of a given TanCAR (39).

#### 2.2.1 Preclinical studies evaluating TanCAR-T cells

In a study by Zah and colleagues, CD19 x CD20 TanCAR-T cells outperformed single-targeted CD19.CAR-T cells in terms of cytotoxic activity after co-culture with WT (CD19^+^CD20^+^) or CD19-knockout (KO) Raji cells. TanCAR-T cells retained their effector functions even after repeated antigen stimulation. Moreover, they showed remarkable levels of tumor control in immunodeficient mice engrafted with WT or a combination of WT plus CD19^-^ Raji cells (40).

In solid tumors, Grada et al. tested CD19 x HER2 TanCAR-T cells against the HER2^+^ Daoy medulloblastoma cell line modified to artificially express the CD19 antigen. The induction of CD19 expression on target cells resulted in an exponential enhancement in TanCAR-T cell killing and interferon gamma (IFN-γ) secretion. Importantly, TanCAR-T cells retained their cytolytic capabilities in the context of HER2 downregulation on target cells. In a xenograft mouse model of medulloblastoma, CD19 x HER2 TanCAR-T cells significantly prolonged the survival of treated mice, particularly after the induction of CD19 expression on target cells (41).

Moreover, patient-derived TanCAR-T cells redirected against HER2 and the α2 subunit of the interleukin (IL)-13 receptor (HER2 x IL13Rα2 TanCAR-T cells) were shown to induce higher levels of cytolysis against autologous glioblastoma multiforme (GBM) cells compared to single targeted HER2.CAR- or IL13Rα2.CAR-T cells, dual CAR-T cells co-expressing the anti-HER2 and anti- IL13Rα2 CARs, or a mixture of HER2.CAR- and IL13Rα2.CAR-T cells (pooled CAR-T cells). TanCAR-T cells also showed a significantly enhanced cytokine production and a more sustained antitumor activity after co-culture with the U373 GBM cell line. Notably, concomitant HER2 and IL13Rα2 binding was found to induce a synergistic effect on cytokine production by TanCAR-T cells. Mice engrafted with GBM cells and treated with TanCAR-T cells had a significantly prolonged survival compared to those receiving single-targeted CAR-T cells. This next-generation CAR platform also allowed to counteract antigen escape. Indeed, tumors relapsing after TanCAR-T cell infusion did not show complete antigen loss, but rather maintained low levels of expression of both the HER2 and IL13Rα2 antigens (42).

Interestingly, TanCARs can be repurposed for the simultaneous targeting of malignant cells and elements of the tumor microenvironment (TME). This could be especially useful to disrupt the complex interplay between tumor cells and bystander elements, which has been shown to contribute to cancer growth and invasiveness (43). Alberti et al. designed CD33 x CD146 TanCAR-cytokine-induced killer (CIK) cells to concomitantly target CD33^+^ AML blasts and CD146^+^ bone marrow (BM) mesenchymal stromal cells (44). The aim was to counteract the pro-leukemic and immunosuppressive effects of the BM niche mediated, at least in part, by this specific stromal component (45–47).

#### 2.2.2 Clinical trials evaluating TanCAR-T cells

As for the clinical translation of the TanCAR system, the NCT03019055 phase I/Ib study evaluated CD20 x CD19 TanCAR-T cells in subjects with R/R CD19^+^ or CD20^+^ B-cell cancers. Investigators reported an overall response rate of 82% at day 28 from initial infusion, with a 64% CR rate. Among the 12 patients who received the CAR-T cell dose chosen for expansion, the overall response rate was 100%, with 92% of them achieving CRs. Even in primary refractory cases or in those who experienced a subsequent relapse, no evidence of CD19 loss was found. Observed toxicities included grade 3-4 neurotoxicity (in 3 out of 22 patients) and grade 3-4 CRS (in 1 individual) (48).

The NCT03614858 trial assessed the safety and efficacy of CD19 x CD22 TanCAR-T cells in patients with R/R B-ALL. The reported 1 – and 2-year overall survival (OS) rates were 76.2% and 73.8%, respectively. 12 out of 47 patients suffered leukemia relapse, but antigen loss was observed in only 2 of these cases (who relapsed with CD19^-^CD22^+^ disease) (49).

## 3 Improving CAR-T cell safety

To date, the identification of tumor-specific antigens (TSAs) suitable for CAR-T cell targeting has largely remained elusive. Indeed, many TSAs have an intracellular localization, so potential tumor neoepitopes are mainly presented on the cancer cell surface in association with human leukocyte antigen (HLA) molecules (50). Unfortunately, being HLA-unrestricted, conventional CAR-T cells can only recognize cell surface antigens not associated with HLA proteins.

A potential solution could be offered by TCR-CARs or TCR-like CARs. TCR-CARs are composed of a TCR variable (TCRv) domain targeting a peptide-HLA (pHLA) complex, linked to a hinge/transmembrane (H/T) region and an intracytoplasmic tail shared with conventional CARs. TCR-like CARs have the same structure, albeit with an anti-pHLA scFv instead of an anti-pHLA TCRv. This allows to avoid possible rearrangements between the native and artificial TCR αβ chains, abating the risk of autoimmune toxicities resulting from the generation of autoreactive TCRv fragments (51).

However, it should be noted that most neoepitopes are different between patients, requiring personalized scFv or TCRv designs for each individual patient. Moreover, HLA molecules are frequently downregulated by malignant cells as an immune escape mechanism, hindering their recognition by TCR – or TCR-like CARs (52).

In the next sections, we will focus on on-target-off-tumor toxicities caused by conventional CAR-T cells targeting TAAs. A notable example is the prolonged B-cell aplasia observed in patients treated with CD19.CAR-T cells, due to the expression of CD19 on normal B lymphocytes. While this specific toxicity is manageable with Ig replacement therapy, the same does not hold true in the context of other hematological as well as solid tumors. For instance, targeting the CD33 myeloid marker in AML can produce an intolerable myelosuppression, making it necessary to proceed to allogeneic hematopoietic stem cell transplantation (alloHSCT) in all treated patients (53). Severe adverse events attributable to off-tumor effects were also reported in clinical trials involving patients with metastatic solid tumors: evidence of hepatotoxicity was observed in a clinical study evaluating carbonic anhydrase-IX (CAIX).CAR-T cells for the treatment of metastatic renal cell carcinoma. This was attributed to off-tumor effects against CAIX^+^ bile duct epithelial cells (54). Moreover, a patient with lung metastases from colon cancer suffered fatal acute respiratory distress syndrome (ARDS) after the infusion of HER2.CAR-T cells, due to off-tumor effects against HER2^dim^ lung epithelial cells (55).

**Figure 2:**
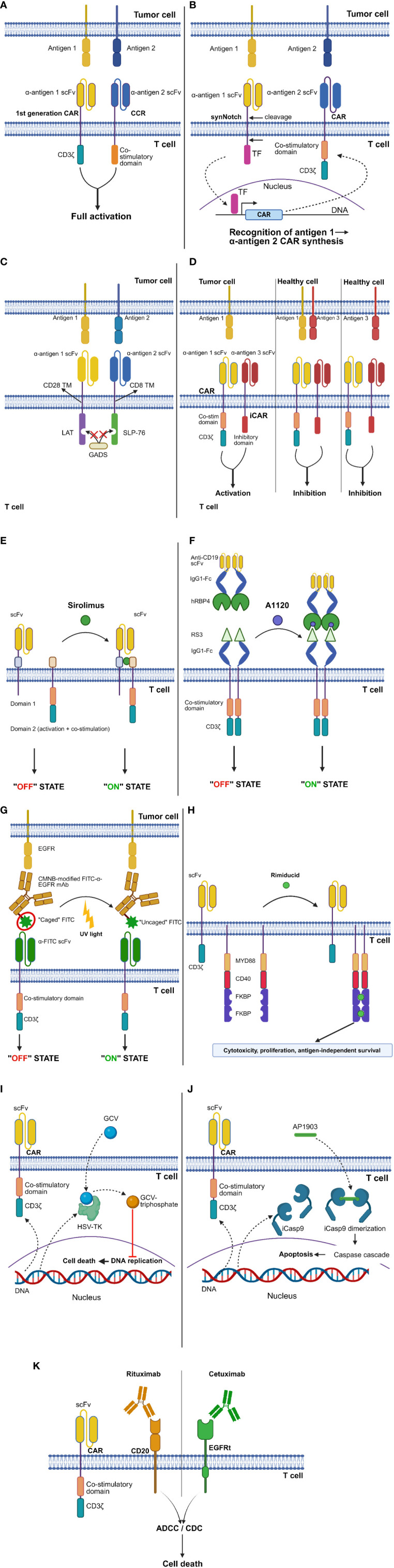
CAR design strategies to improve safety. **(A)** “AND” logic-gating: “combinatorial antigen recognition with balanced signaling”. **(B)** “AND” logic-gating: synthetic Notch (synNotch) platform. **(C)** “AND” logic-gating: logic-gated intracellular network (LINK) system. **(D)** “NOT” logic-gating: inhibitory CAR (iCAR) strategy. **(E)** ON-switches: dimerizing agent-regulated immunoreceptor complex (DARIC) platform. **(F)** ON-switches: human retinol binding protein 4 (hRBP4)-RS3 switch. **(G)** ON-switches: light-controlled switch. **(H)** ON-switches: GoCAR-T^®^ platform. **(I)** OFF-switches: Herpes simplex virus thymidine kinase (HSV-TK) switch. **(J)** OFF-switches: inducible caspase 9 (iCasp9) switch. **(K)** OFF-switches: truncated epidermal growth factor receptor (EGFRt)-cetuximab and CD20-rituximab switches. See Glossary for abbreviations. Modified from Refs. 7, 56–60. Figure created with BioRender.com.

Therefore, considerable research efforts have been directed towards improving CAR-T cell safety profile (**Figure 2**).

### 3.1 Transient CAR expression

A relatively straightforward approach could entail the induction of a transient CAR expression by delivering the corresponding transgene via mRNA electroporation (61). The key advantage of a short-lived CAR expression lies in the rapid termination of any possible off-tumor effects. In addition, RNA CAR-T cell manufacturing is more rapid and cost-effective compared to viral vector- or transposon-based approaches. This technique also allows to transduce T lymphocytes with multiple constructs, thereby broadening their specificity. Due to the transient nature of CAR expression, a drawback of this transfection approach could lie in the induction of weaker and less sustained antitumor effects by RNA CAR-T cells. However, this could potentially be overcome by repeated CAR-T cell administration.

#### 3.1.1 Clinical trials evaluating RNA CAR-T cells

Disappointing results in terms of efficacy have been reported in a phase I trial of mRNA-engineered CD123.CAR-T cells in patients with R/R CD123^+^ AML (NCT02623582) (62). This resulted in the early termination of the study and prompted the investigators to employ a lentiviral system to achieve a stable CD123 CAR expression. Therefore, a separate clinical study involving lentivirally transduced CD123.CAR-T cells has been planned.

In another trial, transient responses were observed in subjects with R/R Hodgkin lymphoma (HL) treated with mRNA-electroporated CD19.CAR-T cells, without severe toxicities (NCT02277522) (63).

Lastly, in a phase I study of mRNA-engineered MSLN.CAR-T cells for the treatment of metastatic ductal adenocarcinoma of the pancreas (NCT04809766), disease stabilization was observed in 2 out of 6 treated patients. The total metabolic active tumor volume substantially decreased in one patient, with a radiological CR in liver metastases even in the absence of effects on the primary tumor. No cases of CRS or dose-limiting toxicities were reported (64).

### 3.2 “AND” – and “NOT” logic-gated CAR-T cells

As mentioned above, logic-gating can also be exploited to improve the safety profile of CAR-T cells. Specifically, the “AND” – and “NOT” gating strategies have been developed to mitigate on-target-off-tumor toxicities (7, 11, 39).

#### 3.2.1 “AND” logic gating

In a 2013 study, Kloss and colleagues introduced a novel approach called “combinatorial antigen recognition with balanced signaling” (38). This method involves splitting the T-cell signaling and co-stimulatory domains, typically combined *in cis* in the traditional CAR design, into 2 separate chimeric receptors. The latter target different antigens and are delivered together to T lymphocytes, often in a single construct. One of these receptors is usually a first-generation CAR, while the other is termed a “chimeric co-stimulatory receptor” (CCR). The CCR is characterized by an intracellular portion comprising only co-stimulatory domains, such as CD28 and 4-1BB. The aim is to elicit a full-blown cytotoxic response by CAR-T cells only against double-positive but not single antigen-expressing cells, due to the lack of the “second signal” required for CAR-T cell activation in the latter scenario (**Figure 2A**). However, the achievement of true cancer specificity requires a low-affinity scFv as the extracellular domain of the first-generation CAR, since CARs bearing only the CD3ζ intracellular moiety are still able to elicit T-cell cytolytic functions. In the above-mentioned study, CAR-T cells simultaneously expressing a first-generation, high-affinity anti-CD19 CAR and an anti-PSMA CCR eradicated not only double positive (CD19^+^PSMA^+^) cancers, but also CD19^+^PSMA^-^ ones. Instead, the administration of CAR-T cells with an affinity-tuned anti-PSCA CAR and an anti-PSMA CCR resulted in the eradication of only double-positive (PSCA^+^PSMA^+^) prostate cancer cells. Interestingly, neoplasms expressing only the antigen recognized by the low-affinity first-generation CAR (PSCA^+^PSMA^-^) were spared even in mice concomitantly engrafted with double positive (PSCA^+^PSMA^+^) tumor cells in a separate anatomical location. This confirms that T-cell activation remains limited to the double-positive tumor niche (38).

Perriello et al. exploited the “AND”-gating approach in AML by engineering CIK cells with a CD33.CCR and a low-affinity IL-3 zetakine recognizing the CD123 antigen. This “AND”-gated product was highly cytolytic against AML cell lines *in vitro* and significantly prolonged the survival of immunodeficient mice engrafted with the KG-1 AML cell line. No significant toxicities against CD33^+^CD123^+^ hematopoietic stem cells (HSCs) and CD123^+^ endothelial cells were observed *in vitro* (65).

The synthetic Notch (synNotch) platform is a modular “AND”-gating strategy based on the basic framework of Notch signaling (66). Upon the recognition of a specific target molecule via its antigen-binding portion, the synNotch receptor undergoes cleavage in its intracellular domain, releasing a transcription factor that promotes the synthesis of a CAR directed against a different antigen (**Figure 2B**). This allows to prevent T-cell activation against tissues expressing either one of the two target antigens, while a complete cytotoxic response is elicited upon the encounter of double-positive tumor cells. Morsut et al. first demonstrated the feasibility of replacing the extracellular and intracellular portions of the WT Notch receptor with specific protein domains to drive novel intercellular signaling cascades (67). The same research group showed that primary T cells equipped with an anti-green fluorescent protein (GFP) synNotch receptor, whose intracellular domain can induce the transcription of an anti-CD19 CAR, undergo activation only after co-culture with target cells expressing both the designated antigens. This spatially controlled CAR synthesis was confirmed *in vivo* by generating xenograft mouse models simultaneously bearing WT (CD19^+^GFP^-^) and modified (CD19^+^GFP^+^) Daudi tumor cells (in separate body sites). Engineered T cells showed evidence of CAR expression in the CD19^+^GFP^+^ but not in the CD19^+^GFP^-^ Daudi tumor. Compared to non-transduced T lymphocytes, synNotch-CAR-T cells significantly prolonged the survival of mice with CD19^+^GFP^+^ but not CD19^+^GFP^-^ tumors. Due to the relatively short half-life of CAR expression after cessation of the synNotch signal, CAR-T cells primed by GFP^+^ tumors did not show any significant cytotoxic activity against single-positive CD19 tumors established in a separate body site. Thus, *in vivo* T-cell activation remained confined to the double-positive tumor (68).

synNotch receptors can also be designed to recognize peptide-HLA complexes, thereby allowing engineered T cells to respond to intracellular antigens. Williams et al. transduced primary T cells with an anti-HLA-A2 – α-fetoprotein (AFP) synNotch controlling the transcription of an anti-HER2 CAR. This synthetic circuit allowed for the selective killing of double-positive but not of single-positive targets (69).

A potential limitation of the synNotch system, which is shared with other “AND” logic-gating strategies, is that the downregulation of either or both target antigens could allow tumors to escape CAR-T cell-mediated killing. However, the same research group proved that the synNotch platform can be used to design more complex cellular recognition circuits. Specifically, they conceived a 3-input “AND”-gating system, designed either in series or in parallel. The in-series circuit was obtained by engineering T cells with an anti-EGFR synNotch activating the synthesis of an anti-hepatocyte growth factor receptor (anti-MET) synNotch, in turn controlling the transcription of a HER2.CAR. As for the in-parallel circuit, the first (anti-EGFR) synNotch activated the expression of a secreted adapter composed of an anti-HER2 scFv linked to a peptide neo-epitope (PNE), while the second (anti-MET) synNotch induced the synthesis of a PNE.CAR. Whereas the in-series circuit induced the selective killing of only triple-positive targets, the in-parallel circuit demonstrated a cytotoxic activity also against dual-positive EGFR^+^HER2^+^ cells. This was ascribed to antigen-independent activation of the anti-MET synNotch, leading to the synthesis of the PNE.

Lastly, they showed that it is possible to combine different forms of logic gating in a single cellular recognition circuit. To this end, they engineered T cells with an anti-GFP synNotch receptor activating the expression of a conventional CD19.CAR, in combination with an anti-HER2 synNotch receptor controlling the synthesis of the proapoptotic factor truncated BH3-interacting domain death agonist (tBID). This “AND-NOT” circuit spared HER2^+^ target cells irrespective of their GFP and/or CD19 expression, due to the activation of the so-called OFF-Notch. HER2^-^GFP^-^CD19^+^ target cells were also spared, since the absence of the GFP antigen prevented CD19.CAR synthesis. On the other hand, HER2^-^GFP^+^CD19^+^ targets were efficiently killed (69).

A peculiar form of “AND”-gating strategy recently proposed by Tousley et al. is the Logic-gated Intracellular NetworK (LINK) system (**Figure 2C**). This approach is unique in that it leverages intracellular mediators involved in the TCR signaling cascade (i.e., linker for activation of T cells [LAT] and SH2 domain-containing leukocyte protein of 76 kDa [SLP-76]) to design CARs devoid of the CD3ζ intracellular domain. Specifically, T lymphocytes were engineered with two chimeric receptors directed against separate antigens and bearing either the LAT or the SLP-76 intracellular domains, with the aim to completely abolish CAR-T cell activation against single antigen-expressing targets. However, T lymphocytes continued to respond to single-positive cells due to the heterodimerization of LAT and SLP-76 mediated by the GRB2-related adaptor downstream of Shc (GADS) protein. Therefore, targeted mutations were strategically introduced into the LAT and SLP-76 coding sequences to disrupt GADS binding, resulting in an absolute degree of tumor-selective killing. The LINK platform was found to outperform other “AND” logic-gating systems, such as the synNotch strategy, in terms of both *in vivo* antitumor activity and safety. Indeed, undesired toxicities were observed in mice treated with synNotch – or conventional CAR-T cells but not in those receiving LINK-CAR-T cells (56).

##### 3.2.1.1 Clinical trials evaluating “AND” logic-gated CAR-T cells

The NCT06186401 phase I trial is being conducted to assess the safety and efficacy of synNotch CAR-T cells in patients with EGFRvIII^+^ recurrent GBM. Specifically, this platform consists of an anti-EGFRvIII synNotch receptor activating the transcription of a TanCAR directed against IL13Rα2 and ephrin type-A receptor 2 (EphA2) (70). The rationale behind this study is based on preclinical research indicating that IL13Rα2 x EphA2 TanCAR-T cells show significantly greater antitumor activity compared to single-targeted IL13Rα2.CAR – or EphA2.CAR-T cells, both *in vitro* and in murine xenograft models (71).

#### 3.2.2 “NOT” logic gating

Under physiological conditions, T-cell activation is tightly controlled by a balance between stimulatory and inhibitory signals. The latter derive, for example, from immune checkpoint receptors like programmed cell death protein 1 (PD-1) and cytotoxic T lymphocyte-associated protein 4 (CTLA-4) (72). On these grounds, a “NOT”-gating strategy has been proposed that entails the engineering of T cells with a conventional CAR plus a separate chimeric receptor, known as “inhibitory CAR” (iCAR). The latter recognizes a different antigen and bears the intracellular portion of the PD-1 or CTLA-4 molecule in its cytoplasmic tail. In this way, the engagement of the iCAR ligand (I) results in CAR-T cell inhibition, even in cases where the antigen targeted by the conventional CAR (A) is present on the same target cell. CAR-T cell activation is thus observed only against A^+^I^-^ tissues (**Figure 2D**). A remarkable drawback of the iCAR approach is that it only allows to generate monospecific cellular products. This, in turn, poses the risk of antigen-negative tumor relapses.

Fedorov and colleagues demonstrated that the artificial expression of an anti-CD19 CAR and an anti-PSMA iCAR in primary T cells significantly impairs their cytotoxic potential, cytokine secretion, and proliferative abilities in a co-culture with CD19^+^PSMA^+^ artificial antigen-presenting cells (AAPCs). On the other hand, no detrimental effects on iCAR-T cell effector functions are seen against targets lacking the antigen recognized by the iCAR (i.e., CD19^+^PSMA^-^ AAPCs). This selective inhibition of CAR-T cell function was confirmed in a xenograft mouse model, where iCAR-T cells eradicated CD19^+^PSMA^-^ but not double-positive B-cell tumors. Of note, iCAR engagement did not permanently impair the functional properties of engineered T lymphocytes: even after being challenged with CD19^+^PSMA^+^ AAPCs, iCAR-T cells showed similar levels of cytotoxicity, cytokine production, and proliferation as compared to conventional CD19.CAR-T cells in a subsequent co-culture with CD19^+^PSMA^-^ AAPCs (73).

The “NOT”-gating approach has also been employed in the setting of AML, allowing to target the leukemic stem cell (LSC) marker CD93 (74) while sparing CD93^+^ healthy endothelial cells. Richards et al. engineered T cells to express a conventional CD93.CAR in combination with anti-CD19 iCARs. CD93.CAR-T cells bearing a PD-1-based iCAR showed a significantly lower cytokine secretion compared to CD93.CAR-T cells when challenged with CD93^+^ endothelial cells modified to co-express CD19. Importantly, these “NOT”-gated CAR-T cells demonstrated preserved cytokine release upon co-culture with the THP-1 AML line (75).

Other “NOT”-gated platforms leverage the loss of heterozygosity (LOH) for the HLA-A*02 antigen, which frequently occurs in malignant cells but not in healthy tissues. For instance, the A2B694 “NOT” logic-gated platform (developed by A2 Biotherapeutics, Inc., Westlake Village, CA, USA) is composed of a third generation MSLN.CAR and an anti-HLA-A*02 iCAR bearing the leukocyte immunoglobulin-like receptor (LIR)-1 intracellular domain. This allows for the selective eradication of MSLN^+^ tumor cells showing HLA-A*02 LOH, while sparing MSLN^+^ healthy tissues with preserved HLA-A*02 expression (76).

The basic iCAR design was recently improved by Bangayan and colleagues, who designed iCARs bearing multiple inhibitory domains in their cytoplasmic tail [e.g., PD-1 – PD-1, PD-1 – leukocyte-associated immunoglobulin-like receptor-1 (LAIR-1)]. This novel platform, known as “DiCAR”, outperformed a PD-1-based iCAR system in terms of efficiency. It also produced faster inhibition kinetics, significantly reducing the delay in T-cell inhibition classically observed with first-generation iCARs (77).

##### 3.2.2.1 Clinical trials evaluating “NOT” logic-gated CAR-T cells

As for the clinical translation of “NOT”-gating approaches, the NCT04981119 observational study was conducted to screen subjects with advanced colorectal, pancreatic, or non-small cell lung cancer for HLA-A*02 LOH (78). The aim was to identify patients eligible for a subsequent interventional study (NCT06051695) assessing the above-mentioned A2B694 platform for the treatment of these malignancies. As of early 2024, the NCT06051695 trial is in the recruitment phase (79).

### 3.3 Safety switches

Another strategy to improve the safety of cellular immunotherapies consists in controlling CAR-T cell activation status via the incorporation of safety switches. Specifically, ON-switches promote CAR-T cell transition from an inactive towards a reactive state in a precisely controllable fashion. In contrast, OFF-switches allow for the termination of CAR-T cell activity in the event of serious toxicities. Several classes of safety switches have been developed. Here, we will focus mainly on small molecule-based systems.

#### 3.3.1 ON-switches

The dimerizing agent-regulated immunoreceptor complex (DARIC) is an ON-switch system whereby the intracellular portion and antigen-binding region of conventional CARs are split into 2 separate proteins co-expressed by engineered T cells (**Figure 2E**). Therefore, even in the presence of target antigen-expressing cells, CAR-T cells remain functionally inert. The supplementation of the mammalian target of rapamycin (mTOR) inhibitor sirolimus results in the dimerization of the two DARIC proteins, restoring CAR-T cell responsiveness (“ON” state). In the presence of sirolimus, CD19.DARIC-CAR-T cells were shown to mediate potent antigen-dependent cytolysis against the Nalm-6 ALL line both *in vitro* and *in vivo.* Only minimal cytotoxicity was observed against the same ALL line in the absence of sirolimus. Of note, the cytotoxicity kinetics were found to be similar between CD19.DARIC-CAR-T cells and conventional CD19.CAR-T cells (57).

Zajc and colleagues designed an ON-switch system composed of human retinol binding protein 4 (hRBP4) in combination with a binder derived from an archaeal protein (RS3). In this system, the binding affinity between its 2 components is greatly increased in the presence of the small molecule A1120. The authors exploited this ON-switch platform to finely regulate CAR-T cell activation: primary T cells were transduced with a CAR whose extracellular portion consisted of the above-mentioned binder linked to an IgG1-Fc spacer domain. The other component of the system (“chain II”) was the hRBP4 protein linked to an anti-CD19 scFv (**Figure 2F**). In the concomitant presence of chain II and A1120, ON-switch CAR-T cells were found to be comparable to conventional CD19.CAR-T cells in terms of cytokine release and antigen-specific cytotoxicity against the Nalm-6 ALL line. However, when A1120 was not added to the co-culture, the antileukemic activity and cytokine production of ON-switch CAR-T cells became similar to those of control T cells, even in the presence of chain II (58).

Besides small molecule-controlled systems, light-operated switches have also been developed. Kobayashi et al. covalently modified FITC-labeled anti-EGFR mAbs with 5-carboxymethoxy-2-nitrobenzyl (CMNB) caging groups, to hinder FITC recognition by FITC.CAR-T cells (**Figure 2G**). The latter effectively killed EGFR^+^ targets in the presence of uncaged FITC-anti-EGFR mAbs. On the other hand, they failed to exert significant cytotoxicity and to release effector cytokines when combined with CMNB-modified (“caged”) FITC-anti-EGFR mAbs.

UV light exposure restored FITC.CAR-T cell reactivity against EGFR^+^ cells in the presence of CMNB-modified mAbs, thus acting as an inducible and controllable switch. Importantly, no cellular damage due to UV light was reported (59).

The applicability of this ON-switch system could be hindered by poor tissue penetrance of UV light. However, this limitation could be overcome by employing advanced light-delivering devices such as optical-lens microneedle arrays (80).

The GoCAR-T^®^ platform employs a peculiar type of ON-switch, whereby T lymphocytes are transduced with a first-generation CAR and an inducible MyD88/CD40 (iMC) molecule. The latter is a membrane protein composed of truncated CD40 and truncated MyD88 fused with 2 FK506 binding protein (FKBP) domains, which induce iMC dimerization in the presence of the small molecule rimiducid (**Figure 2H**). This platform is unique in that GoCAR-T cells are not completely dependent on the ON-switch to exert their effector functions (since they express a fully functional first-generation CAR). Rather, the ON-switch boosts GoCAR-T cell activation by providing them with co-stimulatory signals.

Foster et al. demonstrated that iMC dimerization results in enhanced proliferation, persistence, and antitumor efficacy of PSCA.CAR-T cells against PSCA^+^ pancreatic cancer *in vivo*. In addition, iMC activation was shown to induce antigen-independent CAR-T cell survival. This could be especially useful, e.g., to promote CAR-T cell engraftment in the context of a low tumor burden or to prolong CAR-T cell persistence after tumor eradication (60).

Similarly to AdCAR-T cells, a potential limitation of ON-switches is that the concentration of the switch activator in the tumor niche needs to be maintained above a certain threshold. In addition, it should be noted that the activation kinetics of the various ON-switches are different. Therefore, a delayed ON-switch system engagement could potentially hinder effective tumor control.

##### 3.3.1.1 Clinical trials evaluating ON-switch platforms

The safety and efficacy of the BPX-601 anti-PSCA GoCAR-T^®^ platform (developed by Bellicum Pharmaceuticals, Inc., Houston, TX, USA) have been evaluated in patients with metastatic prostate cancer (NCT02744287). Rimiducid administration was found to induce CAR-T cell expansion in the peripheral blood of the 8 treated patients, accompanied by a rapid rise of serum cytokine levels. Early reports indicated encouraging results in terms of biological activity, with 1 PR and 3 cases of stable disease. As for the safety profile of this platform, CRS occurred in all patients, while ICANS was observed in 2 subjects. In addition, one case of neutropenic sepsis with possible hemophagocytic lymphohistiocytosis was reported (81). However, the subsequent occurrence of grade IV CRS in a patient enrolled in the study led to the discontinuation of the trial. The company also halted enrollment in a separate trial evaluating the BPX-603 anti-HER2 GoCAR-T^®^ platform in patients with advanced HER2^+^ solid tumors (NCT04650451) (82, 83).

#### 3.3.2 OFF-switches

Among OFF-switches, the Herpes simplex virus thymidine kinase (HSV-TK) system allows to achieve CAR-T cell depletion upon ganciclovir (GCV) administration. The underlying mechanism involves GCV phosphorylation by HSV-TK, leading to the generation of GCV-triphosphate nucleotide analogues that interfere with DNA synthesis (**Figure 2I**). This suicide switch has mainly been evaluated in the context of donor lymphocyte infusion to patients who relapsed after T cell-depleted BM transplantation, with the aim to control possible graft-versus-host disease (GvHD) (84). Despite its validated efficacy, however, it should be noted that this T-cell depleting strategy has a relatively slow onset of action. Also, HSV-TK is potentially immunogenic.

A safety switch with no immunogenicity and a more rapid onset of action is the inducible caspase 9 (iCasp9) system, which leverages the intrinsic apoptotic pathway (85). Specifically, the iCasp9 gene encodes a fusion molecule which also contains a ligand-binding portion. In this way, it is possible to induce Casp9 dimerization and CAR-T cell apoptosis upon the intravenous administration of the AP1903 ligand (86) (**Figure 2J**).

Minagawa and colleagues showed that the retroviral transduction of activated T cells (ATCs) with a construct encoding the iCasp9 and an anti-CD33 CAR allows to efficiently redirect T lymphocytes against CD33^+^ AML cells, including pAML samples. Importantly, a sizeable proportion of iCasp9-CD33.CAR-T cells was depleted following the supplementation of an iCasp9 activator, with only negligible effects on untransduced ATCs or CD33.CAR-ATCs. The degree of iCasp9-CAR-T cell depletion was nearly complete following the co-supplementation of BCL inhibitors or the alkylating agent mafosfamide (87).

Hoyos et al. demonstrated that iCasp9-CD19.CAR-T cells are efficiently depleted upon supplementation of a small molecule activator of iCasp9. Moreover, *in vivo* experiments revealed that these modified T lymphocytes are significantly superior to CD19.CAR-T cells in terms of antitumor activity and expansion potential upon antigen encounter (88).

CAR-T cell depletion can also be achieved by leveraging Ab-dependent cell-mediated cytotoxicity (ADCC) and complement-dependent cytotoxicity (CDC). The CD20-rituximab and truncated epidermal growth factor receptor (EGFRt)-cetuximab suicide switches represent two possible ways of implementing this safety strategy. Specifically, T cells are transduced with a construct containing the desired CAR and either the CD20 or the modified EGFR gene. This allows to induce apoptotic cell death (via ADCC) or complement-mediated lysis (via CDC) of engineered T lymphocytes by administering an anti-CD20 or anti-EGFR mAb, respectively (**Figure 2K**).

Philip et al. showed that T lymphocytes transduced with a suicide gene combining epitopes from the CD20 and CD34 antigens are selectively eliminated *in vivo* upon rituximab administration (89).

Key advantages offered by the CD20 and EGFRt switches are the rapid elimination of CAR-T cells and the possibility of employing clinically available mAbs. However, these systems are heavily dependent on the biodistribution of the corresponding mAbs. Their clinical translation could also be hindered by on-target toxicities (e.g., against CD20^+^ healthy cells) and other adverse events associated with mAb administration (90).

An *in vitro* comparison of different suicide gene systems revealed that the iCasp9, CD20, and HSV-TK platforms have similar efficacy in achieving T-cell depletion. However, there were significant differences in the kinetics of cell death induction, with the first 2 switches reaching full effect much faster than the HSV-TK system (91).

A shared limitation of suicide switches is that they are reactive rather than preventative measures, since they can only be deployed after the onset of undesired toxicities. Moreover, these systems deplete CAR-T cells irrespective of their therapeutic efficacy, since they do not allow to discriminate between desirable antitumor responses and unwanted off-tumor effects. Consequently, the timing of suicide switch activation becomes a crucial consideration for clinicians, adding an additional layer of complexity to therapeutic decision-making.

##### 3.3.2.1 Clinical trials evaluating OFF-switch platforms

Among the clinical trials involving suicide gene switches, the NCT03016377 phase I/II study assessed the safety and efficacy of autologous iCasp9-CD19.CAR-T cells in patients with R/R B-cell malignancies. In this trial, the administration of rimiducid, a protein dimerizer that acts as an iCasp9 activator, was found to alleviate steroid- and tocilizumab-refractory ICANS (92).

iCasp9-CD19.CAR-T cells were also evaluated in an academic, phase I/II trial enrolling pediatric patients with B-ALL or B-cell NHL. CRS occurred in 58.8% of patients, but reached grade 3 only in one subject, and no life-threatening adverse events requiring iCasp9 safety switch activation were noted (93).

Del Bufalo et al. performed an academic, phase I/II trial (NCT03373097) to evaluate iCasp9-CAR-T cells redirected against disialoganglioside GD2 in children and young adults with R/R, high-risk neuroblastoma. The incidence of CRS was 74% (20 of 27 patients), but most cases were mild and safety switch activation was needed only in a single subject (94).

The NCT02028455 phase I/II trial assessed the use of CD19.CAR-T cells expressing the EGFRt safety gene in 45 children and young adults with R/R B-ALL. No cases of acute, life-threatening toxicities requiring cetuximab administration were reported (95).

In the NCT02159495 phase I study evaluating EGFRt-CD123.CAR-T cells in patients with CD123^+^ R/R AML or BPDCN, no cases of severe CRS or neurotoxicity requiring cetuximab administration were observed (96).

### 3.4 Targeting tumor-specific membrane protein glycoforms

Even if the expression of a given membrane protein is shared between healthy and cancerous cells (as is the case for TAAs), the latter could still express a different isoform of said protein. This could be due to specific post-translational modifications (e.g., glycosylation) occurring in malignant but not in healthy tissues. On these premises, some research groups have focused on the identification of tumor-specific membrane protein glycoforms that could allow to selectively target neoplastic cells without causing damage to vital tissues. This could represent a promising strategy to design safer cellular immunotherapies without having to resort to complex logic-gating or suicide gene systems.

In several cancer types, the membrane mucin MUC1 is frequently expressed as the Tn (GalNAca1-O-Ser/Thr)-glycoform, which is absent in healthy tissues. On these grounds, Posey Jr. and colleagues demonstrated that CAR-T cells redirected against Tn-MUC1 induce the lysis of Tn-MUC1^+^ cells while sparing various MUC1^+^ healthy human tissues *in vitro*. Their efficacy was confirmed in a xenograft model of T-cell leukemia, where Tn-MUC1.CAR-T cells significantly prolonged the survival of mice when compared to unmanipulated T lymphocytes or CD19.CAR-T cells. Interestingly, Tn-MUC1.CAR-T cells were able to distinguish Tn-MUC1^+^ from Tn-MUC1^-^ cells within the same co-culture well, thus confirming the absence of off-target effects.

The shared expression of the Tn-MUC1 glycoform across several cancer types could enable Tn-MUC1.CAR-T cells to recognize and kill multiple different tumors while maintaining a favorable safety profile. Indeed, they were found to outperform unmanipulated T lymphocytes and CD19.CAR-T cells in terms of antitumor activity in a xenograft mouse model of pancreatic cancer (97).

#### 3.4.1 Clinical trials on targeting tumor-specific protein isoforms

The NCT05239143 phase I trial enrolling patients with advanced epithelial tumors is evaluating the safety and efficacy of the P-MUC1C-ALLO1 platform (developed by Poseida Therapeutics, Inc., Kansas City, MO, USA). The latter is an allogeneic CAR-T cell product directed against the MUC1-C epitope, which is selectively expressed in carcinomas of the lung, esophagus, colon, breast, and ovary. Early reports indicated a favorable safety profile of this platform, with no dose-limiting toxicities, GvHD, or CRS observed in the first 3 treated patients (98).

### 3.5 Deletion or modification of CAR target antigens

A novel and potentially revolutionary approach to prevent off-tumor effects consists in ablating the expression or modifying the structure of the CAR target antigen in healthy cells. This could be especially useful in the context of hematological malignancies, where the modification of HSCs could allow to combine CAR-T cell therapy with HSCT in the absence of on-target myelotoxicity against healthy hematopoietic precursors.

#### 3.5.1 Target antigen deletion on normal cells

Kim and colleagues exploited the clustered regularly interspaced short palindromic repeats (CRISPR) – CRISPR-associated protein 9 (Cas9) technology to delete the CD33 gene from normal human HSC, with the aim to make them resistant to CD33-directed therapies. The authors demonstrated that primary human CD34^+^ cells (i.e., immature hematopoietic progenitors) with disrupted CD33 expression are comparable to control human HSCs in terms of engraftment and differentiation capabilities when inoculated into immunodeficient mice. CD33-KO cells did not show any alterations in terms of morphology or functional capabilities (e.g., cytokine secretion, generation of reactive oxygen species, phagocytosis, etc.), both *in vitro* and *in vivo*. Also, no changes in gene expression profile or in the differentiation pattern elicited by the exposure to external factors were noted. These findings were validated in a non-human primate model, where modified HSCs showed preserved engraftment potential and established a morphologically normal hematopoietic process in the BM. Moreover, CD33 KO effectively shielded modified HSCs from CD33.CAR-T cells in a human xenograft model. The absence of bystander toxicities was confirmed by inoculating engrafted mice with the CD33^+^ MOLM-14 AML cell line: CD33.CAR-T cells were able to achieve tumor control in mice engrafted with either CD33-KO – or control HSCs, but caused off-leukemia toxicities only in the latter scenario (99).

Humbert et al. obtained similar results by selectively ablating CD33 exon 2, which codes for the V-set Ig-like domain that is the target of CD33-directed therapies. CD33^ΔE2^ human HSCs showed normal engraftment and differentiation potential in a murine xenograft model and were resistant to the toxic effects of the anti-CD33 Ab-drug conjugate gemtuzumab ozogamicin (100).

#### 3.5.2 Epitope editing

More recently, researchers have managed to incorporate non-synonymous mutations in epitopes of interest to hinder their recognition by CAR-T cells while preserving the function of the corresponding protein (an approach referred to as “epitope editing”). This could allow to target essential molecules without having to abrogate their expression on healthy HSCs.

Marone et al. generated HSCs expressing a modified but fully functional IL-3 receptor alpha-chain (CD123), with the aim to shield them from CD123.CAR-T cells. CD123 is expressed in most AML cases, including on the surface of LSCs. However, this molecule plays a crucial role in the biology of normal HSCs, thus discouraging a KO approach to alleviate on-target toxicities. On the basis of *in silico* mutagenesis studies, the authors engineered two specific amino acid substitutions into HSCs isolated from healthy donors (HDs). In colony-forming assays, epitope-edited HSCs demonstrated comparable differentiation and colony forming potential as WT HSCs. In contrast, CD123-KO HSCs showed a significant competitive disadvantage. Edited HSCs also maintained their engraftment capabilities and long-term repopulating potential when engrafted into immunodeficient mice. Finally, in co-culture assays involving the MOLM-14 AML line and engineered HSCs (either CD123 epitope-edited or CD123-KO), CD123.CAR-T cells depleted malignant cells as well as CD123^hi^ unedited HSCs and CD123^lo^ elements within the CD123-KO HSC population. On the other hand, epitope-edited HSCs were resistant to CAR-T cell cytolysis and retained their expression of the modified CD123 molecule (101).

Casirati et al. optimized a base-editing strategy to introduce desired amino acid variants into the CD123, FMS-like tyrosine kinase 3 (FLT3), and KIT molecules. The aim was to shield these molecules from therapeutic mAbs without altering their function. Epitope-edited HSCs were shown to be fully functional *in vitro* and *in vivo*. In addition, they were resistant to FLT3.CAR-T cells, allowing for the selective elimination of patient-derived AML xenografts (102).

Lastly, since the CD45 antigen is expressed by all nucleated cells of hematopoietic origin (including their malignant counterparts), Wellhausen and colleagues envisioned the use of CD45-epitope editing of HSCs to develop a universal CAR-T cell therapy for blood cancers with no off-tumor toxicities (103).

In all the previously mentioned studies, specificity analyses (e.g., computational off-target prediction) confirmed the safe engineering of HSCs (i.e., no off-target genotoxic effects were detected). However, several concerns still exist regarding gene editing-based cellular therapies. For instance, their manufacturing is highly complex, requiring HSC isolation and genetic manipulation in addition to the conventional CAR-T cell manufacturing process. Therefore, the future clinical translation of these approaches could be hindered by an excessive burden of preparatory procedures imposed on patients, as well as longer manufacturing time and an increased risk of production failures. This, in turn, could have a significant impact on treatment costs and could prevent some patients from receiving CAR-T cell infusions due to disease progression during the prolonged bridging period.

## 4 Optimizing CAR-T cell functionality

As discussed in the previous sections, broadening CAR-T cell specificity and avoiding off-tumor effects are critical for the development of safe and effective cellular immunotherapies. Another aspect that cannot be overlooked entails the optimization of CAR-T cell functional properties to achieve complete and long-lasting tumor control. In this context, several strategies have been proposed, including specific modifications to the basic CAR structure and the modulation of intracellular signaling pathways involved in T-cell antitumor responses.

### 4.1 Choice of co-stimulatory domain(s)

CAR-T cell functional properties are critically dependent on CAR design, as evidenced by the profound differences between CD28 – and 4-1BB – co-stimulated CAR-T cell products in terms of cytolytic activity, cytokine production, memory differentiation, persistence, and metabolic profile. Preclinical studies comparing the antitumor potential of CARs with either a CD28 or a 4-1BB co-stimulatory moiety have yielded mixed results, due in part to the confounding effect associated with the use of different H/T domains (104).

A substantial body of evidence indicates an almost complete overlap between downstream protein phosphorylation events that follow the activation of either CD28 – or 4-1BB-co-stimulated CARs (105). However, some studies highlighted considerable differences in terms of the specific intracellular CAR interaction partners and their relative abundance when comparing CD28 – and 4-1BB – based constructs (106). Therefore, further investigations are needed to more precisely dissect the complex network of pathways that relay CAR-initiated signals. Anyhow, it is widely accepted that phosphorylation events occur more rapidly and with increased magnitude upon the engagement of CD28 co-stimulated CARs (105). This, in turn, is reflected in a more rapid tumor clearance by CD28 – co-stimulated CAR-T cells, both *in vitro* and *in vivo* (107, 108).

Bulk RNA sequencing (RNA-seq) has revealed unique transcriptional programs in 4-1BB – *vs* CD28-CAR-T cells. These differences are observed not only upon T-cell stimulation through the CAR, but also in the resting state, indicating a “tonic signaling signature”. Specifically, 4-1BB – based CAR-T cells tend to be enriched for MHC class II and fatty acid oxidation genes, along with marker genes typical of CD8 central memory (T_CM_)-like cells. CAR engagement amplifies the above-mentioned differences and results also in the upregulation of several cytokine cascades in 4-1BB-CAR-T cells, such as the tumor necrosis factor (TNF)-α, IL-21, and IFN-γ pathways. On the other hand, activated 4-1BB-CAR-T cells downregulate the expression of the PDCD1 gene, resulting in a lower PD-1 surface expression. They also undergo a T helper (Th)1 polarization program, while activated CD28-based products tend to show a Th2 phenotype. The resultant differences in cytokine secretion could influence the risk of CRS after CAR-T cell administration (109).

**Figure 3:**
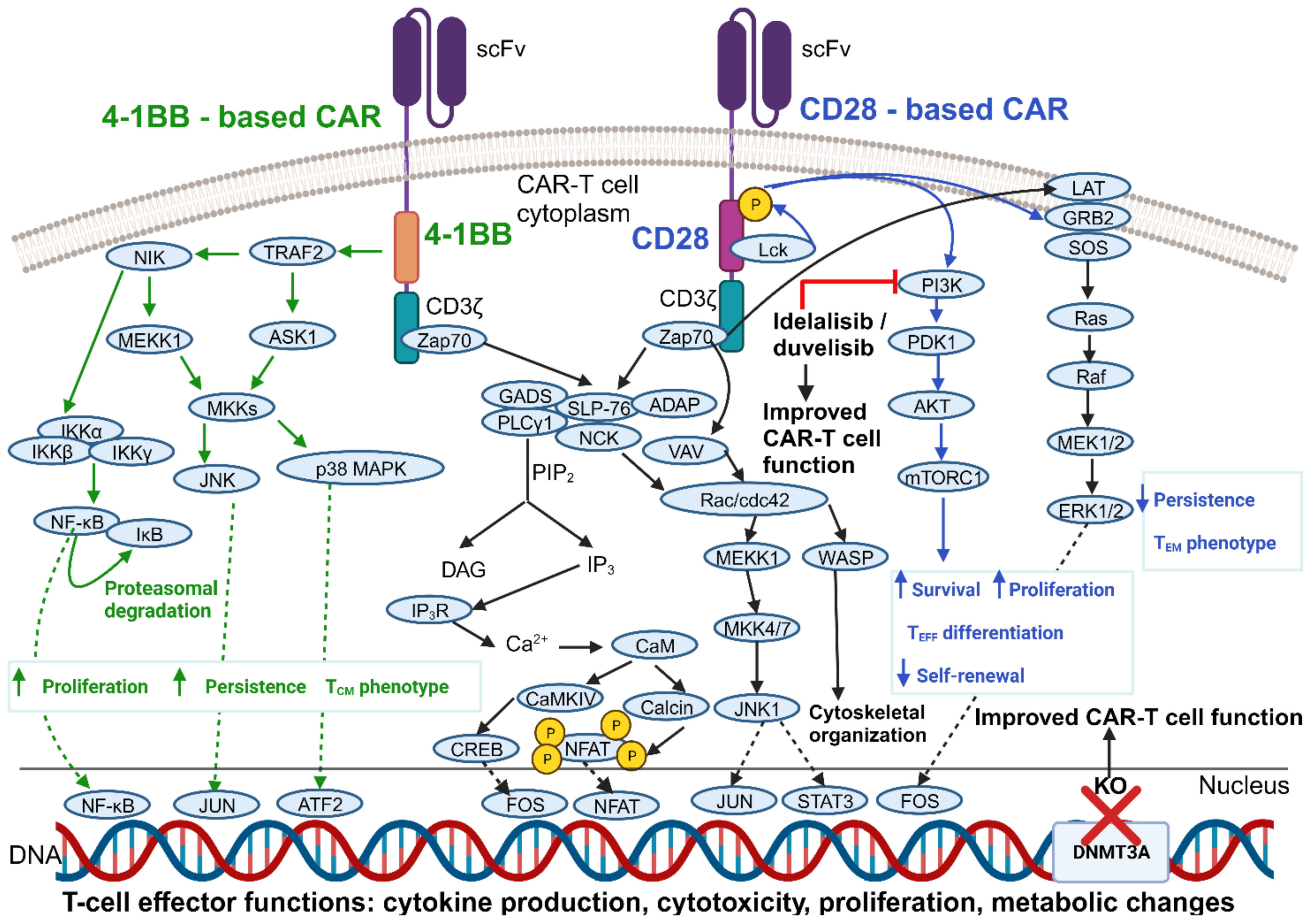
Comparison of 4-1BB – *vs* CD28 – co-stimulated CARs and improvements in CAR-T cell functionality obtained by modulating key intracellular mediators. Shown are the main signaling cascades elicited upon CAR engagement. Only the signaling mechanisms most strongly associated with a given co-stimulatory domain have been reported in its proximity (and color-coded accordingly), although it should be noted that 4-1BB – and CD28 – based CARs likely signal through almost identical pathways. Also shown are strategies to improve CAR-T cell function by leveraging intracellular signaling pathways and gene expression programs. See Glossary for abbreviations. Modified from Ref. 111. Figure created with BioRender.com.

As for *in vivo* persistence, 4-1BB-CAR-T cells generally exhibit longer-lasting anticancer activity compared to CD28 co-stimulated products. This may be due to a slower tumor eradication process, leading to extended interactions between CAR-T and malignant cells. Another factor contributing to the enhanced persistence of 4-1BB-CAR-T cells is their relative resistance to functional exhaustion during prolonged antigen stimulation. This is evidenced by their increased cytokine production and reduced expression of exhaustion markers (e.g., lymphocyte-activation gene 3 [LAG-3], T-cell immunoglobulin and mucin domain 3 [TIM-3]) as compared to CD28-CAR-T cells (110) (**Figure 3**).

#### 4.1.1 Clinical trials evaluating CD28 – or 4-1BB – based CAR-T cells

To this day, there is insufficient clinical evidence to establish the superiority of either 4-1BB – or CD28 – co-stimulated CARs in terms of safety and anticancer efficacy. This is due, at least in part, to differences in several other factors besides the co-stimulatory domains (e.g., CAR design, CAR-T cell manufacturing processes, conditioning chemotherapy protocols, etc.) that complicate possible comparisons between different clinical trials (104).

Among FDA- and EMA-approved products targeting CD19, tisagenlecleucel (tisa-cel) and lisocabtagene maraleucel (liso-cel) are lentivirally-transduced and contain a 4-1BB co-stimulatory domain, but differ in their CAR H/T domains and T-cell expansion protocols (112). On the other hand, the CD28 – co-stimulated products, namely brexucabtagene autoleucel (brexu-cel) and axicabtagene ciloleucel (axi-cel), are both retrovirally-transduced, contain identical H/T domains, and undergo the same manufacturing process. Moreover, lymphodepleting chemotherapy preceding 4-1BB – or CD28-CAR-T cell administration consists in the same cyclophosphamide-fludarabine combination regimen, except in the case of tisa-cel.

Bearing in mind the limitations outlined above, the CR rates and remission duration observed in R/R B-ALL patients treated with 4-1BB – or CD28 – co-stimulated CD19.CAR-T cells are similar. Interestingly, however, consolidative alloHSCT confers an event-free survival (EFS) benefit only in subjects treated with 4-1BB – based products. In the case of R/R B-cell NHL, 4-1BB-based liso-cel and CD28-based axi-cel are associated with comparable CR and 12-month progression-free survival (PFS) rates, which are superior to those achieved with 4-1BB – based tisa-cel.

4-1BB – and CD28 – co-stimulated CAR-T cells also differ in terms of their safety profile, since the occurrence of CRS and ICANS has been reported more frequently in clinical studies evaluating CD28-based products (104, 112–122) (**Table 1**).

**Table 1:** Efficacy and safety of CD28 - or 4-1BB - based CD19. CAR-T cells in select trials enrolling patients with B-cell malignancies.

Disease. Patient number and characteristics	Co-stimulatory domain	CR rate	PFS	CRS rate; grade ≥3 CRS rate	Neurotoxicity rate; grade ≥3 neurotoxicity rate	Trial (Ref.)
B-ALL, NHL. 20 children and young adults*	CD28	70%*	78,8% of MRD-negative CRs ongoing at a median follow-up of 10 mos*	76%; 29%*	20%; 5%*	Lee et al., 2015 (114)
B-ALL. 25 children and young adults	CD28	75%	N/A	80%; 16%	72%; 28%	Curran et al., 2019 (115)
B-ALL. 25 children and young adults; 5 adults	4-1BB	90%	70% of CRs ongoing at a median follow-up of 7 mos	100%; 27%	43%; N/A	Maude et al., 2014 (116)
B-ALL. 14 children and young adults	4-1BB	86%	42% of CRs ongoing at 14 mos	93%; 0%	50%; 7%	Ghorashian et al., 2019 (117)
DLBCL (77 patients), PMBCL (8), tFL (16). Adults	CD28 (axi-cel)	54%	42% at 15 mos	93%; 13%	64%; 28%	Neelapu et al., 2017 (118)
DLBCL (14 patients), FL (3), MCL (2), BL (1). Adults	CD28	55%	40% at 17 mos	85%; 10%	20%; 5%	Brudno et al., 2020 (119)
DLBCL (22 patients), MCL (4), FL (6). Adults	4-1BB	33%**	N/A	N/A; 13%	N/A; 28%	Turtle et al., 2016 (112)***
DLBCL (251 patients), PMBCL (15), FL (3). Adults	4-1BB (liso-cel)	53%	44% at 12 mos	42%; 2%	30%; 10%	Abramson et al., 2020 (120)

Axi-cel, axicabtagene ciloleucel; B-ALL, B-cell acute lymphoblastic leukemia; BL, Burkitt lymphoma; CR, complete remission; CRS, cytokine release syndrome; DLBCL, diffuse large B-cell lymphoma; FL, follicular lymphoma; liso-cel, lisocabtagene maraleucel; MCL, mantle-cell lymphoma; mos, months; N/A, not applicable; NHL, non-Hodgkin lymphoma; PFS, progression-free survival; PMBCL, primary mediastinal B-cell lymphoma; tFL, transformed follicular lymphoma.

*B-ALL cohort. **50% in patients receiving lymphodepletion with cyclophosphamide/fludarabine. ***CAR-T cells administered in a CD4^+^/CD8^+^ ratio of 1:1. Modified from Ref. 104.

### 4.2 Manipulation of CAR-T cell activation threshold and target antigen density

Optimal CAR-T cell performance can only be achieved when target antigen density exceeds a certain threshold. Therefore, manipulating the antigen density requirement for efficient CAR-T cell responses has emerged as a promising strategy to boost CAR-T cell functionality. Alternatively, CAR-T cell reactivity could be enhanced by inducing target antigen upregulation on the surface of malignant cells.

#### 4.2.1 Tuning the antigen density requirement for CAR-T cell reactivity

Majzner et al. investigated the impact of the CAR H/T region on the antigen density threshold for CAR-T cell activation. They showed that the presence of a CD28 – instead of a CD8α-H/T domain significantly enhances the antitumor function of 4-1BB-based CD19.CAR-T cells against CD19^lo^ B-cell leukemia. Indeed, H/T-modified 4-1BB-CAR-T cells performed similarly to CD28-based products, with the remarkable advantage of a more durable persistence. This improvement in 4-1BB-CAR-T cell function against antigen^lo^ targets was confirmed in murine models of solid tumors, including neuroblastoma and osteosarcoma. The underlying mechanism was found to involve an increased CAR clustering and recruitment of downstream signaling molecules upon antigen encounter (123). These findings could allow to counteract one of the most prevalent resistance mechanisms exploited by cancer cells, namely antigen modulation resulting in antigen^lo^ relapse.

#### 4.2.2 Promoting target antigen upregulation on cancer cells

Priming cancer cells with the hypomethylating drugs decitabine (DAC) or azacitidine (AZA) could represent a relatively straightforward way to promote CAR target antigen upregulation, thus enhancing CAR-T cell antitumor activity (124).

Li et al. showed that pretreatment of B-cell NHL lines with DAC at concentrations that have negligible effects on malignant cell viability results in a significantly increased CD19 expression. This, in turn, rendered malignant cells more susceptible to CD19.CAR-T cell cytolysis. Importantly, no significant impairment in CAR-T cell functional properties was reported after DAC exposure (125).

A similar strategy was employed by El Khawanky and colleagues, who demonstrated that AZA treatment increases CD123 expression on the surface of AML cell lines and renders them more immunogenic. This, in turn, facilitates their lysis by CD123.CAR-T cells. AZA administration was also found to influence the subpopulation composition of CAR-T cells infused into AML xenograft mouse models. Specifically, the authors reported an increase in the proportion of CTLA-4^-^ CAR-T cells, which were found to possess superior functional capabilities in terms of malignant cell killing and cytokine production. This ultimately led to a significant prolongation of the survival of AZA-pretreated mice compared with the control group (126).

Similarly, AZA was found to increase CD70 expression on the surface of AML cells, both *in vitro* and *in vivo*. AZA pretreatment of AML-bearing mice before CD70.CAR-T cell administration resulted in enhanced tumor control and improved survival compared to non-pretreated mice (127).

##### 4.2.2.1 Clinical trials evaluating strategies to promote target antigen upregulation

The NCT03196830 phase II trial assessed the role of a DAC-based lymphodepletion regimen preceding CD19 x CD22 TanCAR-T cell infusion in patients with R/R diffuse large B-cell lymphoma (DLBCL). Investigators reported a CR rate of 63,6% at a median follow-up of 10.9 months, and a 2-year OS of 54.3%. Toxicities were mostly mild and reversible (128).

### 4.3 Modulation of cell-intrinsic signaling pathways and gene expression programs

A growing body of evidence indicates that leveraging T-lymphocyte intrinsic signaling pathways and gene expression programs could represent another way to enhance CAR-T cell functionality (**Figure 3**).

#### 4.3.1 Phosphoinositide 3-kinase signaling pathway inhibition

The phosphoinositide 3-kinase (PI3K) signaling pathway is a master regulator of T-cell activation upon antigen encounter, promoting the proliferation, survival, and differentiation of activated immune cells (129).

Previous research has shown that the PI3K inhibitor idelalisib can modulate T-cell differentiation in patients with B-cell NHL (130). Idelalisib also improved the persistence and antitumor function of adoptively transferred CD8^+^ T cells *in vivo* (131, 132). On this basis, investigators have explored the use of PI3K inhibitors to induce a more favorable differentiation profile and enhance the antitumor function of CAR-T cells. Funk and colleagues showed that supplementation of the PI3Kδ/γ inhibitor duvelisib (Duv) to CAR-T cell cultures induces epigenetic modifications characteristic of stem-like cells and upregulates co-stimulatory receptor – and TCR-activated signaling pathways. This translates into an improved cytolytic potential of Duv-CAR-T cells *in vitro* and *in vivo*. Indeed, in a murine CLL model, CD19.Duv-CAR-T cells demonstrated superior expansion, persistence, tumor control, and resistance to exhaustion as compared to conventional CAR-T cells (133).

Similar findings were reported by Petersen et al., who showed that idelalisib supplementation improves the expansion potential of CD5.CAR-T cells and boosts their antitumor functionality against lymphoma cells *in vitro* and *in vivo* (134).

#### 4.3.2 DNA methyltransferase 3A deletion

A key limitation of CAR-T cell therapy, especially in solid tumors, is their functional exhaustion due to chronic antigen stimulation. This dysfunctional state appears to be subjected, at least in part, to epigenetic regulation. Zebley and colleagues showed that, after infusion into B-ALL patients, CD19.CAR-T cells acquire DNA methylation programs linked to functional exhaustion. These programs are maintained even in the absence of sustained antigenic stimulation. As a result, CAR-T cell ability to respond to a subsequent tumor relapse is impaired. Therefore, modulating DNA methylation programs could represent a promising strategy to promote sustained tumor control by CAR-T cells (135).

DNA methyltransferase 3A (DNMT3A) is a key enzyme in the establishment of *de novo* DNA methylation programs (136). Given that *Dnmt3a* deletion in mouse T cells can prevent their exhaustion during chronic viral infections (137), researchers have explored the possibility of deleting DNMT3A in CAR-T cells to preserve their antitumor functionality.

In an *in vitro* model of chronic stimulation developed by Prinzing et al., CAR-T cells lacking DNMT3A (DNMT3A KO) showed enhanced antigen-specific expansion compared to conventional CAR-T cells. Their cytokine response upon repeated antigen encounter was similar to that of control CAR-T cells, except for a remarkable IL-10 upregulation. This cytokine was found to support DNMT3A KO CAR-T cell survival and proliferation during chronic antigen exposure by limiting sustained co-stimulatory signaling. As a result, DNMT3A KO CAR-T cells showed preserved antigen-specific cytolysis upon repeated antigen stimulation. They also displayed a reduced expression of the TIM-3 exhaustion marker and a predominantly T_CM_ phenotype. The favorable properties of DNMT3A KO CAR-T cells reported *in vitro* translated to an improved and more sustained antitumor efficacy in murine models of glioma and osteosarcoma (138).

## 5 Conclusion and future perspectives

CAR-T cells have reshaped the treatment landscape of B-cell malignancies, offering new hope for individuals with an otherwise dismal prognosis. At present, however, not all patients benefit from this therapeutic modality to the same degree, owing to limitations of the specific CAR design employed or to functional defects of adoptively transferred cells.

By introducing rational modifications into the CAR design, researchers have managed to improve CAR-T cell selectivity towards malignant tissues and mitigate the risk of toxicities associated with the targeting of TAAs. The choice of specific co-stimulatory domains, the manipulation of healthy hematopoietic cells targeted by CAR-T cell activity, and the modulation of T-lymphocyte intrinsic intracellular signaling cascades represent additional advances in the field.

Nonetheless, there are still several outstanding issues which need to be addressed, especially in the context of solid tumors. Such bottlenecks pertain not only to the intrinsic properties of CAR-T cells, but also involve their complex interactions with malignant cells and other immunosuppressive elements of the TME (139). Another key area of improvement could be related to the optimization of bridging and conditioning regimens preceding CAR-T cell infusion, to provide a more favorable environment for CAR-T cell engraftment (140).

As we continue to develop our understanding of the factors underlying therapeutic success, we will manage to design safer and more effective cellular immunotherapies. The anticipated goal is to induce long-lasting remissions, and possibly cure, in an ever-growing number of patients.

## Author contributions

AA: Conceptualization, Writing – original draft, Writing – review & editing. MB: Writing – review & editing. EG: Writing – review & editing. AB: Supervision, Writing – review & editing, Funding acquisition, Resources. MS: Writing – review & editing, Conceptualization, Funding acquisition, Resources, Supervision. ST: Writing – review & editing, Supervision, Conceptualization, Writing – original draft.

## Glossary

**Table:**

4-1BBL	4-1BB ligand
AAPC	artificial antigen-presenting cell
Ab	antibody
ADAP	adhesion and degranulation-promoting adapter protein
AdCAR	adapter chimeric antigen receptor
ADCC	antibody-dependent cell-mediated cytotoxicity
AFP	α-fetoprotein
AKT	Akt protein kinase
ALL	acute lymphoblastic leukemia
alloHSCT	allogeneic hematopoietic stem cell transplantation
AM	adapter molecule
AML	acute myeloid leukemia
AMP	adenosine monophosphate
ARDS	acute respiratory distress syndrome
ASK1	apoptosis signal-regulating kinase 1
ATC	activated T cell
ATF2	activating transcription factor 2
axi-cel	axicabtagene ciloleucel
AZA	azacitidine
BBIR	biotin-binding immune receptor
BiAb	bispecific antibody
BiTE	bispecific T-cell engager
BL	Burkitt lymphoma
BM	bone marrow
BPDCN	blastic plasmacytoid dendritic cell neoplasm
brexu-cel	brexucabtagene autoleucel
Ca^2+^	calcium ion
CAIX	carbonic anhydrase-IX
Calcin	calcineurin
CaM	calmodulin
cAMP	cyclic AMP
CAMKIV	calcium/calmodulin-dependent protein kinase IV
CAR	chimeric antigen receptor
Cas9	CRISPR-associated protein 9
Casp	caspase
CCR	chimeric costimulatory receptor
CD	cluster of differentiation
CDC	complement-dependent cytotoxicity
CDR	complementarity-determining region
CIK	cytokine-induced killer
CLL	chronic lymphocytic leukemia
CLL-1	C-type lectin-like molecule-1
CMNB	5-carboxymethoxy-2-nitrobenzyl
CR	complete remission
CR_i_	complete remission with incomplete hematologic recovery
CREB	cAMP response element-binding protein
CRISPR	clustered regularly interspaced short palindromic repeats
CRS	cytokine release syndrome
CTLA-4	cytotoxic T lymphocyte-associated protein 4
DAC	decitabine
DAG	diacylglycerol
DARIC	dimerizing agent-regulated immunoreceptor complex
DL	dose level
DLBCL	diffuse large B-cell lymphoma
DNMT3A	DNA methyltransferase 3A
Duv	duvelisib
EFS	event-free survival
EGFR	epidermal growth factor receptor
EGFRt	truncated epidermal growth factor receptor
EGFRvIII	epidermal growth factor receptor variant III
EMA	European Medicines Agency
EpCAM	epithelial cell adhesion molecule
EphA2	ephrin type-A receptor 2
ERK1/2	extracellular signal-regulated kinase 1/2
Fc	fragment crystallizable
FDA	Food and Drug Administration
FITC	fluorescein isothiocyanate
FKBP	FK506 binding protein
FL	follicular lymphoma
FLT3	FMS-like tyrosine kinase 3
FOS	protein c-Fos
GADS	GRB2-related adaptor downstream of Shc
GalNAcα1-O-Ser/Thr	N-acetylgalactosamine linked to a serine or threonine residue
GBM	glioblastoma multiforme
GCV	ganciclovir
GFP	green fluorescent protein
GRB2	growth factor receptor-bound protein 2
GvHD	graft-versus-host disease
HD	healthy donor
HER2	epidermal growth factor receptor 2
HL	Hodgkin lymphoma
HLA	human leukocyte antigen
hRBP4	human retinol binding protein 4
HSC	hematopoietic stem cell
HSCT	hematopoietic stem cell transplantation
HSV-TK	Herpes simplex virus thymidine kinase
H/T	hinge/transmembrane
ICANS	immune effector cell-associated neurotoxicity syndrome
iCAR	inhibitory chimeric antigen receptor
iCasp9	inducible caspase 9
IFN-γ	interferon-γ
Ig	immunoglobulin
IκB	inhibitor of κB
IKK-	inhibitor of κB kinase-
IL-	interleukin-
iMC	inducible MyD88/CD40
IP_3_	inositol 1,4,5-trisphosphate
IP_3_R	inositol 1,4,5-trisphosphate receptor
JNK	c-Jun N-terminal kinase
JUN	Jun transcription factor
kDa	kilodalton
KO	knockout
LAG-3	lymphocyte-activation gene 3
LAIR-1	leukocyte-associated immunoglobulin-like receptor-1
LAT	linker for activation of T cells
Lck	lymphocyte-specific protein tyrosine kinase
LINK	logic-gated intracellular network
LIR-1	leukocyte immunoglobulin-like receptor-1
liso-cel	lisocabtagene maraleucel
LOH	loss of heterozygosity
LSC	leukemic stem cell
mAb	monoclonal antibody
MAPK	mitogen-activated protein kinase
MCL	mantle-cell lymphoma
MCSP	melanoma-associated chondroitin sulfate proteoglycan
MEK1/2	mitogen-activated protein kinase kinase 1/2
MEKK1	mitogen-activated protein kinase kinase kinase 1
MET	hepatocyte growth factor receptor
MKK	mitogen-activated protein kinase kinase
mos	months
MRD	minimal residual disease
MSLN	mesothelin
mTOR	mammalian target of rapamycin
mTORC1	mTOR complex 1
N/A	not applicable
NCK	non-catalytic region of tyrosine kinase
NF-κB	nuclear factor kappa-light-chain-enhancer of activated B cells
NFAT	nuclear factor of activated T cells
NHL	non-Hodgkin lymphoma
NIK	NF-κB-inducing kinase
ORR	objective response rate
OS	overall survival
P	phosphate group ([PO_4_]^3-^)
p38 MAPK	p38 mitogen-activated protein kinase
pAML	primary acute myeloid leukemia
PCR	polymerase chain reaction
PD-1	programmed cell death protein 1
PDK1	phosphoinositide-dependent kinase-1
PFS	progression-free survival
pHLA	peptide-HLA
PI3K	phosphoinositide 3-kinase
PIP_2_	phosphatidylinositol 4,5 bisphosphate
PLCγ1	phospholipase C, gamma 1
PMBCL	primary mediastinal B-cell lymphoma
PNE	peptide neoepitope
PR	partial remission
PSCA	prostate stem cell antigen
PSMA	prostate-specific membrane antigen
qPCR	quantitative PCR
qRT-PCR	quantitative reverse transcription PCR
Rac/cdc42	Ras-related C3 botulinum toxin substrate/cell division control protein 42 homolog
Raf	rapidly accelerated fibrosarcoma protein
Ras	rat sarcoma protein
RNA-seq	RNA sequencing
R/R	relapsed/refractory
SAR	synthetic agonist receptor
scFv	single-chain variable fragment
SLP-76	SH2 domain-containing leukocyte protein of 76 kDa
SOS	Son of Sevenless
STAT3	signal transducer and activator of transcription 3
STn	sialyl-Tn
synNotch	synthetic Notch
T_CM_	central memory T cell
T_EM_	effector memory T cell
T_N_	naïve T cell
T_SCM_	stem cell-like memory T cell
TAA	tumor-associated antigen
TanCAR	tandem chimeric antigen receptor
tBID	truncated BH3-interacting domain death agonist
TCR	T-cell receptor
TCRv	TCR variable domain
tFL	transformed follicular lymphoma
Th	T helper
TIM-3	T-cell immunoglobulin and mucin domain 3
tisa-cel	tisagenlecleucel
TLR7a	toll-like receptor-7 agonist
TM	target module
TME	tumor microenvironment
TNF-	tumor necrosis factor-
TRAF2	TNF receptor-associated factor 2
TSA	tumor-specific antigen
VAV	Vav guanine nucleotide exchange factor
VH	antibody heavy chain variable domain
VL	antibody light chain variable domain
WASP	Wiskott-Aldrich syndrome protein
WT	wild-type
ZAP-70	zeta-associated protein 70
